# Established and Emerging Regulatory Roles of Eukaryotic Translation Initiation Factor 5B (eIF5B)

**DOI:** 10.3389/fgene.2021.737433

**Published:** 2021-08-27

**Authors:** Prakash Amruth Raj Chukka, Stacey D. Wetmore, Nehal Thakor

**Affiliations:** ^1^Department of Chemistry and Biochemistry, University of Lethbridge, Lethbridge, AB, Canada; ^2^Southern Alberta Genome Sciences Centre (SAGSC), University of Lethbridge, Lethbridge, AB, Canada; ^3^Alberta RNA Research and Training Institute (ARRTI), University of Lethbridge, Lethbridge, AB, Canada; ^4^Canadian Centre of Research in Advanced Fluorine Technologies (C-CRAFT), University of Lethbridge, Lethbridge, AB, Canada; ^5^Department of Biological Sciences, University of Lethbridge, Lethbridge, AB, Canada; ^6^Department of Neuroscience, Canadian Centre for Behavioral Neuroscience (CCBN), University of Lethbridge, Lethbridge, AB, Canada; ^7^Arnie Charbonneau Cancer Institute, Cumming School of Medicine, University of Calgary, Calgary, AB, Canada

**Keywords:** eukaryotic initiation factor 5B (eIF5B), mRNA translation, Non-canonical Translation Ianitiation, IRES, uORF

## Abstract

Translational control (TC) is one the crucial steps that dictate gene expression and alter the outcome of physiological process like programmed cell death, metabolism, and proliferation in a eukaryotic cell. TC occurs mainly at the translation initiation stage. The initiation factor eIF5B tightly regulates global translation initiation and facilitates the expression of a subset of proteins involved in proliferation, inhibition of apoptosis, and immunosuppression under stress conditions. eIF5B enhances the expression of these survival proteins to allow cancer cells to metastasize and resist chemotherapy. Using eIF5B as a biomarker or drug target could help with diagnosis and improved prognosis, respectively. To achieve these goals, it is crucial to understand the role of eIF5B in translational regulation. This review recapitulates eIF5B’s regulatory roles in the translation initiation of viral mRNA as well as the cellular mRNAs in cancer and stressed eukaryotic cells.

## Introduction

Cap-dependent or canonical translation initiation is an intricate process and highly regulated in eukaryotes. It involves multiple initiation factors ranging from small proteins to complex multidomain proteins. The process begins when 5′ cap is recognized by eukaryotic initiation factor 4F (eIF4F) complex and mRNA is recruited onto the 43S preinitiation complex (43S-PIC) (Supplementary Figure 1). Subsequently, this leads to the formation of 48S preinitiation complex (48S-PIC), which scans the 5′ untranslated region (UTR) of the mRNA and recognizes the start codon (AUG). In the final step, eukaryotic initiation factor 5B (eIF5B) promotes the association of small (40S) and large (60S) ribosomal subunits to form elongation competent 80S initiation complex (80S IC) (Supplementary Figure 1).

eIF5B, also known as IF-M2A/hIF2, is a “Arrokoth” shaped GTPase discovered in 1975. It is universally conserved among all eukaryotes and encoded by the *EIF5B* gene (Pestova et al., 2000; Roll-Mecak et al., 2000). eIF5B mediates the association of the 40S and 60S ribosomal subunits during eukaryotic translation initiation (Merrick et al., 1975; Lee et al., 2002; Shin et al., 2002). Although eIF5B is highly conserved, its depletion does not have a substantial effect on cell viability under normal conditions (Lee et al., 2014; Ho et al., 2018; Ross et al., 2019, 2020). Conversely, reduced levels of eIF5B under stress conditions significantly affects cell viability (Lee et al., 2014; Ho et al., 2018).

eIF5B consists of a highly conserved functional C-terminal region (human 587–1220, yeast 397–1,002) and a less conserved N-terminal region (human 1–586, yeast 1–396) (Choi et al., 1998; Lee et al., 1999). Deleting the N-terminal region does not affect cell viability and many *in vitro* studies have shown that N-terminally truncated eIF5B is active (human 587–1,220, yeast 397–1,002) (Pestova et al., 2000; Lee et al., 2002; Shin et al., 2002; Fringer et al., 2007; Pisareva and Pisarev, 2014). On the other hand, the functional C-terminal consists of four domains: G domain (human 629–850, yeast 401–625), domain II (human 856–948, yeast 630–745), domain III (human 951–1,082, 755–855), and domain IV (human 1,076–1,220, yeast 859–1,002) (Pestova et al., 2001; Nag et al., 2016; Huang and Fernandez, 2020). Additionally, domains III and IV are connected by a helix h12 whose deletion in yeast yields non-functional eIF5B similar to the defects observed in ΔeIF5B and Δdomain IV cells (Fringer et al., 2007; Shin et al., 2011).

Studies on eIF5B demonstrated a newer function that parallels the role of eIF2, a Met-tRNA_i_^Met^ delivering eukaryotic initiation factor, during stress conditions (Thakor and Holcik, 2012; Holcik, 2015; Sharma et al., 2016). The mechanism is activated when α-subunit of eIF2 is phosphorylated and sequestered by eIF2B. Under these conditions, eIF5B promotes translation of specific proteins by delivering Met-tRNA_i_^Met^ to the eukaryotic ribosomes, due to its homology to IF-2 which delivers met-tRNAf^Met^ to the bacterial ribosomes (Ross et al., 2019). Additionally, recent studies have unveiled a mechanistic role of eIF5B during the canonical and non-canonical eukaryotic translation initiation (Ross et al., 2019). We and others have also clearly implicated eIF5B in oncogenesis (Wang et al., 2016; Ross et al., 2019; Suresh et al., 2020). Accordingly, this review provides insights into the coordination of eIF5B with other eukaryotic initiation factors to carry out diverse functions during translation initiation in yeast and human cells. This review covers the established roles of eIF5B in 40S ribosome maturation, formation of 48S PIC, stabilization of Met-tRNA_i_^Met^, 60S ribosomal recruitment and 80S complex formation. We also highlight the emerging roles of eIF5B during Met-tRNA_i_^Met^ delivery by coordinating with eIF2A, uORF-mediated translation initiation, and IRES-mediated translation initiation. We further discuss how eIF5B acts as a nexus between non-canonical translation and the survival of cancer cells.

### Role of eIF5B in Pre-40S Ribosome Subunit Maturation

During ribosome biogenesis, the large and small subunits undergo a translation-like cycle where eIF5B mediates the association of the pre-40S and 60S ribosomal subunits, which acts as a quality control step (Lebaron et al., 2012). The resulting complex is not a true 80S initiation-complex (80S IC) as it lacks initiator tRNA and mRNA. As a result, the 60S ribosomal subunit is displaced by the termination factor Rli-1 during 40S subunit maturation (Strunk et al., 2012; Woolford and Baserga, 2013). The 80S-like complex ensures the proper functioning of pre-ribosomes before translation (Strunk et al., 2012). A study on the YKK392 yeast strain devoid of eIF5B has shown a negative effect on the ribosomal subunit association, resulting in the accumulation of pre-40S subunits (Strunk et al., 2012). In general, this accumulation is not detrimental, but delays the formation of the 80S-like ribosomal complex and slows down cell growth (Strunk et al., 2012). Deleting the eIF5B coding gene *FUN12* in yeast also results in the accumulation of pre-18S rRNA, and decreased levels of 27S pre-rRNA and 40S ribosomes (Lebaron et al., 2012; Strunk et al., 2012). Thus, eIF5B is essential for catalyzing the ribosome maturation process in yeast.

### eIF5B Interacts With eIF5 to Stimulate the Formation of 48S Initiation Complex

Human eIF5B and eIF5 synergistically mediate the efficient formation of the 48S initiation complex (48S IC) (Pisareva and Pisarev, 2014). The interaction between domain IV of eIF5B and the C-terminus of eIF5 could be crucial for efficient 48S IC formation (Lin et al., 2018). Affinity studies inferred a higher affinity of eIF5B for eIF5 compared with eIF1A (Lin et al., 2018). It has been hypothesized that human eIF5 and eIF5B together stimulate 43S preinitiation complex (PIC) rearrangement to increase the yield of functional 48S IC (Pisareva and Pisarev, 2014). Additionally, eIF5B deletion results in the destabilization of 48S IC, which was suggested to be induced by eIF5 (Pisareva and Pisarev, 2014). This phenomenon was observed in both optimal and non-optimal AUG context, suggesting the role of eIF5B in 48S IC stabilization (Pisareva and Pisarev, 2014). eIF5 has additional roles as a GTPase-activating protein (GAP) and a GDP-dissociation inhibitor (GDI) (Paulin et al., 2001). While eIF5 induces eIF2-GTP hydrolysis, eIF5B promotes the release of eIF2 from 48S IC (Unbehaun et al., 2004; Pisarev et al., 2006). Furthermore, eIF5B assists in establishing 48S IC on a bona fide AUG and prevents leaky scanning along with eIF5 (Pisareva and Pisarev, 2014; Lin et al., 2018). Since codon scanning and selection during translation initiation are essential for generating functional proteins, these studies collectively provide compelling evidence that eIF5B coordinates with eIF5 to aid the establishment of an efficient 48S IC on a bona fide start codon.

### Met-tRNA_i_^Met^ Stabilization by eIF5B

After eIF2-GTP delivers Met-tRNA_i_ to the P-site of the 40S ribosomal subunit, GAP eIF5 induces eIF2-GTP hydrolysis (Paulin et al., 2001; Majumdar and Maitra, 2005). eIF2B disrupts the eIF5/eIF2-GDP interaction and facilitates eIF2-GDP release (Jennings and Pavitt, 2010; Jennings et al., 2016). In the absence of eIF2, domains III and IV of eIF5B extend into the inter-subunit space and stabilize Met-tRNA_i_^Met^ (Huang and Fernandez, 2020). Conformational changes in domains III and IV facilitate interactions between basic amino acids in domain IV and _7__3_ACCA_76_-Met of Met-tRNA_i_^Met^ (Fernandez et al., 2013). This creates a kink in the Met-tRNA_i_^Met^ stem structure that is not seen in elongation tRNA, which helps Met-tRNA_i_^Met^ simultaneously interact with both mRNA and eIF5B (Wang et al., 2020). Additionally, methionine of tRNA_i_ positions itself in the hydrophobic pocket formed by eIF5B and the uL16 loop of the 60S subunit, and single-molecule experiments suggested that the hydrophobic pocket acts as a residue selectivity filter (Wang et al., 2020). Ultimately, the eIF5B-initiator tRNA complex places the tRNA_i_ aminoacyl end out of the peptidyl transfer center, awaiting GTP hydrolysis. Thus, these eIF5B interactions are vital for stabilization and correct positioning Met-tRNA_i_^Met^ in the initiation complex after eIF2 is displaced from the ribosome, suggesting the absence of eIF5B could delay the transition into elongation.

### Interaction of eIF5B With eIF1A for Ribosome Recruitment

eIF1A is one of the earliest discovered interacting partners of eIF5B. Similar to eIF5B, eIF1A is considered to be a universal translational factor (Sorensen et al., 2001). The interaction between these two initiation factors is thought to be important for eIF5B recruitment and mediating the joining of the large and small ribosomal subunits (Fringer et al., 2007). In contrast, another study proposed that eIF5 recruits eIF5B and eIF1A disrupts their interaction after eIF2-GTP hydrolysis (Lin et al., 2018). In the absence of the ribosome, interactions between eIF5B and eIF1A are disrupted due to intramolecular interactions within each initiation factor (Nag et al., 2016). Interactions between eIF1A and eIF5B are only established after codon recognition when C-terminal tail (CTT) of eIF1A is displaced from the P-site to interact with the adjacent eIF5B (Yu et al., 2009). Domains III and IV of eIF5B interact with the oligonucleotide/oligosaccharide-binding (OB) domain and CTT of eIF1A (Nag et al., 2016). These interactions are critical for the recruitment of the 60S subunit and formation of the 80S complex (Olsen et al., 2003; Acker et al., 2006). Thus, these studies suggest that interactions between the two universally conserved eIF5B and eIF1A initiation factors are necessary to mediate the association of the 40 and 60S subunits by eIF5B, which forms a viable 80S IC.

### Ribosomal Subunit Association Is Expedited by eIF5B

**FIGURE 1:**
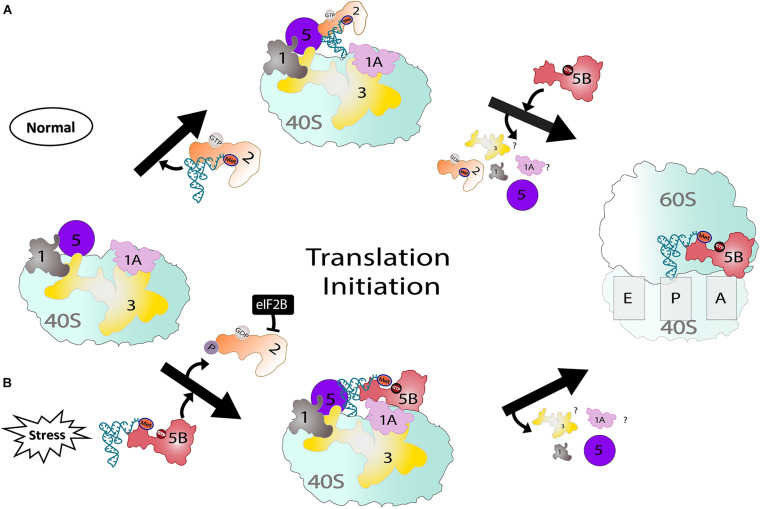
Two pathways showing eIF2-dependent (normal) and eIF5B-dependent translation (stress). **(A)** Under normal conditions, eIF2 delivers Met-tRNA_i_^Met^ to the ribosome and, upon release of eIF2, eIF5B stabilizes Met-tRNA_i_^Met^. Once 48S PIC forms, eIF5B assists in large and small ribosomal subunit joining. **(B)** Under stress conditions, eIF2 is phosphorylated and sequestered by eIF2B. However, eIF5B parallels the role of eIF2 and delivers Met-tRNA_i_^Met^ to the P-site of the 40S ribosome in addition to its usual function during translation.

One primary role of eIF5B is to promote the ribosomal association of the 40S and 60S subunits (Figure 1A; Lee et al., 2002; Shin et al., 2002). The long retention time (30–60 s) of eIF5B on ribosomes indirectly prevents their collision on mRNA before 80S IC transitions into the elongation step (Wang et al., 2019). Apart from ribosomal subunit joining, *in vivo* studies have shown the stabilization of the halfmer polysome (43S PIC + 80S on an mRNA) by eIF5B (Lee et al., 2002). To participate in ribosomal subunit association, eIF5B must be in an active form, which has been proposed to be achieved through a domain release mechanism (Nag et al., 2016). The process begins when the inherently rigid domains III and IV of eIF5B-GDP become flexible upon GTP binding (Kuhle and Ficner, 2014).

Each domain of eIF5B has a specific function during ribosome association: (1) the G-domain interacts with the 60S subunit and is involved in GTP hydrolysis, (2) domain II is anchored to the 40S subunit, (3) domain III also anchors to the 40S subunit, and promotes GTP hydrolysis when Met-tRNA_i_^Met^ is delivered, and (4) domain IV interacts with t-RNA, eIF1A, and eIF5 (Fernandez et al., 2013; Nag et al., 2016; Lin et al., 2018; Huang and Fernandez, 2020). GTP hydrolysis is not required for ribosomal subunit association, but is required for the release of eIF5B and 80S IC transition into the elongation step (Shin et al., 2002; Huang and Fernandez, 2020). If domain III does not recognize a proper Met-tRNA_i_^Met^ delivery or ribosomal association, eIF5B could be trapped in the P/A site, which hampers the recruitment of a new aminoacyl-tRNA, delays the transition to elongation, and obstructs new ribosome recruitment. Although ribosome recruitment occurs even in the absence of eIF5B, 80S IC formation is inefficient and the transition time into the elongation step is longer, resulting in a slow growth phenotype (Fringer et al., 2007; Jiang et al., 2016). Overall, once eIF5B induces the 60S and 40S subunit association, eIF5B-GDP is released, making the 80S initiation complex elongation competent (Fringer et al., 2007).

### eIF5B Interaction With eIF2A in Non-canonical Translation Initiation

When availability of ternary complex (eIF2-GTP-Met-tRNA_i_^Met^) is low under stress conditions, initiation factor eIF2A has been shown to deliver the Met-tRNA_i_^Met^ to the 40S ribosome by coordinating with eIF5B (Kim et al., 2018). eIF5B alone plays a major role in Met-tRNA_i_^Met^ delivery during translation initiation on certain virus mRNAs (Pestova et al., 2008; Yamamoto et al., 2014). *In vitro* studies involving pull down assays suggest that domain IV of eIF5B modestly interacts with the M domain of eIF2A (462–502 aa), but all eIF5B domains are required for a high affinity interaction (Kim et al., 2018). A predicted model suggested that eIF5B domain IV might also be responsible for interacting and delivering Met-tRNA_i_^Met^ (Kim et al., 2018). Unlike eIF2, eIF2A does not depend on GTP to deliver Met-tRNA_i_^Met^, but may rely on GTP hydrolysis by eIF5B for its release from the ribosome (Adams et al., 1975; Zoll et al., 2002; Kim et al., 2018). Studies in *S. cerevisiae* and *Caenorhabditis elegans* showed slow growth phenotype when eIF5B/iffb-1 was knocked down, and the growth deteriorated even more when both eIF5B and eIF2A were knocked down (Zoll et al., 2002; Kim et al., 2018). However, under conditions like hypoxia, the initiation process could be solely eIF5B dependent as depletion of eIF2A has no effect on protein synthesis (Ho et al., 2018). These studies clearly imply that eIF2A augments eIF5B function during Met-tRNA_i_^Met^ delivery, and domain IV of eIF5B that helps stabilize Met-tRNA_i_^Met^ during normal conditions could be indispensable for Met-tRNA_i_^Met^ delivery under stress conditions.

### Role of eIF5B in IRES-Mediated Translation

Internal ribosome entry site (IRES) is a secondary structure present on the mRNA of both viral and cellular origins (Pestova et al., 2008; Thakor and Holcik, 2012; Sharma et al., 2016). These IRES elements can recruit ribosomes directly without a requirement for the 5′-m7G cap during certain stress conditions (Holcik et al., 2000; Thakor and Holcik, 2012; Sharma et al., 2016). Several viral mRNAs contain IRES elements, including but not limited to hepatitis C virus (HCV), classical swine fever virus (CSFV), poliovirus (PV), and coxsackie B virus (CBV) (Pestova et al., 2008; Shatsky et al., 2008; Terenin et al., 2008; Yamamoto et al., 2014). CSFV and HCV IRES in particular depend on eIF5B for translation in the absence of eIF2 (Figure 1B; Pestova et al., 2008; Shatsky et al., 2008; Yamamoto et al., 2014). X-linked inhibitor of apoptosis (XIAP, a caspase inhibitor in eukaryotes) mRNA also contains IRES and its translation is eIF5B dependent upon eIF2 sequestration (Yoon et al., 2006; Thakor and Holcik, 2012; Thakor et al., 2017). In general, XIAP is an important anti-apoptotic protein that plays a substantial role in preventing programmed cell death (Perrelet et al., 2000; LaCasse et al., 2008). Nevertheless, IRES elements of viruses such as cricket paralysis virus (CrPV) do not depend on eIF5B for translation (Deniz et al., 2009; Kerr et al., 2016). Thus, although there is strong evidence that eIF5B is involved in IRES mediated translation of several viral and anti-apoptotic mRNA, not all IRES-containing mRNAs require eIF5B for translation initiation.

### Regulation of uORF Mediated Translation by eIF5B

**FIGURE 2:**
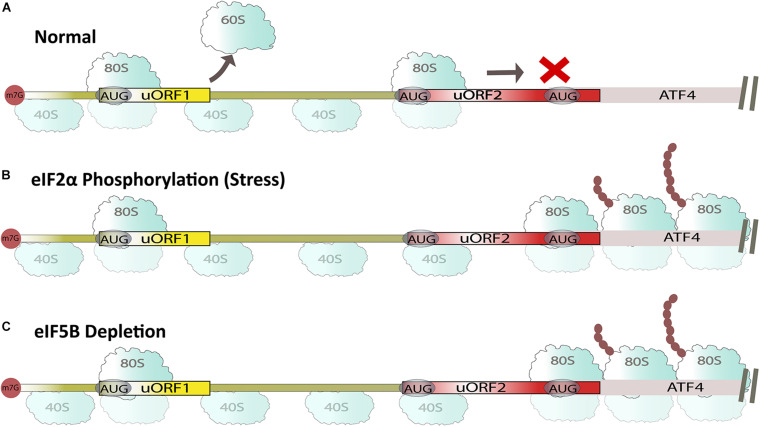
Summary of uORF-mediated ATF4 translation. **(A)** Under non-aberrant normal conditions, ribosomes are recruited on uORF regions, repressing ATF4 expression in the presence of eIF5B. **(B)** Under aberrant stress conditions, ribosomes scan past uORF2, resulting in the synthesis of ATF4 in the presence of eIF5B. **(C)** eIF5B depletion in normal cells also promotes ATF4 synthesis.

Upstream open reading frame (uORF) elements are present in the 5′ untranslated regions (UTR) of mRNA across different species (Chew et al., 2016). These elements inhibit mRNA translation under normal conditions and promote their translation during stress conditions that induce eIF2 phosphorylation (Figures 2A,B; Dever et al., 1992; Barbosa et al., 2013; Pakos-Zebrucka et al., 2016; Ross et al., 2018; Chen and Tarn, 2019). For example, *activating transcriptional factor 4* (*ATF4*) has two uORF regions that keep protein levels low until phosphorylation of α-subunit of eIF2 (Figure 2; Holcik, 2015; Sharma et al., 2016). During *ATF4* mRNA translation under normal conditions, eIF5B prevents leaky mRNA scanning and ribosomes are recruited onto uORFs, which represses the translation of *ATF4* ORF (Ross et al., 2018). In contrast, under stress or eIF5B depleted conditions, ribosomal recruitment occurs on the main ORF AUG sequence, causing de-repression of *ATF4* mRNA translation (Figure 2C; Ross et al., 2018). This in turn leads to the upregulation of other proteins like C/EBP homologous protein (CHOP) and growth arrest and DNA damage-inducible protein (GADD34) (Pakos-Zebrucka et al., 2016). This was further corroborated by our recent study on human embryonic kidney 293T (HEK-293T) cells, where depleting eIF5B induced ER stress upregulating mRNA and protein levels of CHOP and GADD34 (Bressler et al., 2021). eIF5B requires cooperativity with initiation factors eIF5 and eIF1A to repress ATF4 expression, and the mechanism of repression is predominantly dependent on uORF2 (Ross et al., 2018). Apart from ATF4, general control non-depressible 4 (GCN4), a transcription factor containing 4 uORFs, is repressed both in starved and non-starved yeast cells in the absence of eIF5B (Shin et al., 2002; Murakami et al., 2018). Similarly, knocking down eIF5B decreased the levels of programmed death-ligand 1 (PD-L1) in heme starved non-small cell lung cancer (NSLC) and lewis lung carcinoma (LLC) cells (Suresh et al., 2020). In contrast, eIF5B negatively regulates p21 and p27 in serum starved THP1 cells, which have been linked to cell cycle progression, and anti-apoptosis (Lee et al., 2014). Numerous uORF dependent proteins have been identified whose expression is regulated by eIF5B, and many of these proteins have implications in cancer resistance and cell cycle. Thus, regulating eIF5B could impact such pathways necessary for cancer survival.

### eIF5B Promotes Cancer Cell Survival

Previous studies suggest eIF5B mediates the IRES containing subset of mRNA translation to resist apoptosis in cancer cells (Ross et al., 2019). Translation of IRES-containing mRNAs, which encode anti-apoptotic proteins such as XIAP, Bcl-xL, and Cellular Inhibitor of Apoptosis Protein 1 (cIAP1) as well as Nuclear factor erythroid 2-related factor 2 (Nrf2), are regulated by eIF5B in glioblastoma multiforme (GBM) cells (Ross et al., 2019, 2020). There is also growing evidence of high eIF5B expression levels in various malignancies like GBM, lung adenocarcinoma (LUAD) and hepatocellular carcinoma (HCC), signifying the importance of eIF5B as the stress-related tumorigenic eIF (Wang et al., 2016; Ross et al., 2019; Suresh et al., 2020). In fact, high eIF5B levels were associated with poor prognosis for HCC patients, while low eIF5B levels resulted in smaller tumor sizes, lower vascular invasions, and better survival rates (Wang et al., 2016). Studies on GBM cells show eIF5B depletion leads to reduced cell growth due to inhibition of the NF-κB pathway and sensitization to temozolomide (TMZ)-mediated apoptosis (Ross et al., 2019, 2020). Further, eIF5B aids the survival of GBM cells under hypoxic conditions by acting as one of the essential translational factors for the synthesis of hypoxia-response proteins and regulates carbon metabolism (Ho et al., 2018). In HCC, eIF5B indirectly promotes metastasis and proliferation by upregulating ArfGAP with SH3 Domain, Ankyrin Repeat and PH Domain 1 (ASAP1) expression both *in vivo* and *in cellulo* (Wang et al., 2016). Depletion of eIF5B in maraba virus infected and uninfected U2OS cells resulted in reduced Bcl-xL expression at both the transcriptional and translational levels (Hassanzadeh et al., 2019). This suggests eIF5B plays a role in regulating apoptosis during oncolytic virus treatment (Hassanzadeh et al., 2019). Depletion of eIF5B reduced tumor mass and propagation in lewis lung carcinoma (LLC) and non-small cell lung cancer (NSLC) cell lines, respectively, demonstrating the dependence of cancer cells on eIF5B for growth and proliferation (Suresh et al., 2020). In line with these findings, high levels of eIF5B under heme depletion induce translation of integrated stress response (ISR) dependent PD-L1, which inhibits T-cell activity. This clearly indicates that eIF5B promotes the survival of LLC and NSLC malignancies (Suresh et al., 2020). Additionally, under serum deprivation in the THP1 cell line, an acute increase in eIF5B levels is observed (Lee et al., 2014). When eIF5B is depleted, global translation is reserved and early G0 phase prohibition occurs, indicating the regulatory role of eIF5B in the cell cycle (Lee et al., 2014). eIF5B also regulates developmental pathways, in particular the mammalian target of rapamycin (mTOR) and mitogen-activated protein kinase (MAPK) pathways, which are activated by epidermal growth factor receptor (EGFR) (Jiang et al., 2016). Thus, these studies highlight that supressing the activity of eIF5B disrupts many pathways, alluding to its importance in oncogenesis.

Considering its pivotal role in cancer cells, eIF5B is emerging as a therapeutic target for cancer treatment. Numerous small molecules and proteins have been identified that could inhibit the activity of eIF5B. For example, a small molecule denoted LWW31 was suggested to inhibit eIF5B activity and lower the viability of cancer cells (Wu et al., 2016). Similarly, ribavirin triphosphate, a guanosine triphosphate analog, has been hypothesized to inhibit the activity of eIF5B (Galmozzi et al., 2012). Additionally, proteins like Puf6p and HIV-1 matrix have been identified, which have shown to repress the function of eIF5B in yeast and human, respectively (Wilson et al., 1999; Deng et al., 2008). These promising works highlight that further research is required to screen and identify clinically relevant small molecules and peptides for targeting eIF5B in “hard-to-treat” and “high-fatality” cancers such as GBM.

## Concluding Remarks

The research works summarized in this review clearly illustrate that eIF5B is a crucial factor for canonical translation initiation. Its role in uORF- and IRES-mediated translation initiation has also been unequivocally established. To this end, the ability of eIF5B to interact with eIF5, eIF2A, and eIF1A as well as to bind and deliver initiator tRNA is critical for mRNA translation. eIF5B is overexpressed in several malignancies and its aberrant expression has been linked to glioblastoma, lung carcinoma, and hepatocellular carcinoma. Due to egregious levels of eIF5B and its conspicuous role in non-canonical translation, eIF5B is an important initiation factor for oncogenesis. It would be ideal to target eIF5B with the goal to regulate non-canonical translation using new small molecules. To achieve this goal, multi-omics (transcriptome, metabolome, as well as translatome and proteome) studies in cancer models are required to establish eIF5B as a biomarker for certain types of cancer. Additionally, fundamental biomedical and pre-clinical studies are necessary to establish eIF5B as a therapeutic target for cancer treatments. Finally, drug discovery research that includes the integration of computer-aided drug design (CADD) with machine learning and is complimented by traditional wet bench experiments must be done. Cancers with upregulated eIF5B have been challenging to treat, and this could change if druggability of eIF5B is further explored as it is a viable therapeutic target.

## Author Contributions

NT proposed the idea for mini-review. PC wrote the manuscript. NT and SW edited the manuscript and assisted with the figure concepts. All authors contributed to the article and approved the submitted version.

## Conflict of Interest

The authors declare that the research was conducted in the absence of any commercial or financial relationships that could be construed as a potential conflict of interest.

## Publisher’s Note

All claims expressed in this article are solely those of the authors and do not necessarily represent those of their affiliated organizations, or those of the publisher, the editors and the reviewers. Any product that may be evaluated in this article, or claim that may be made by its manufacturer, is not guaranteed or endorsed by the publisher.
