# Supplementary figures and images for: Established and Emerging Regulatory Roles of Eukaryotic Translation Initiation Factor 5B (eIF5B)
Source: Front Genet. 2021 Aug 27;12:737433. doi: 10.3389/fgene.2021.737433 (PMC8430213; doi:10.3389/fgene.2021.737433)

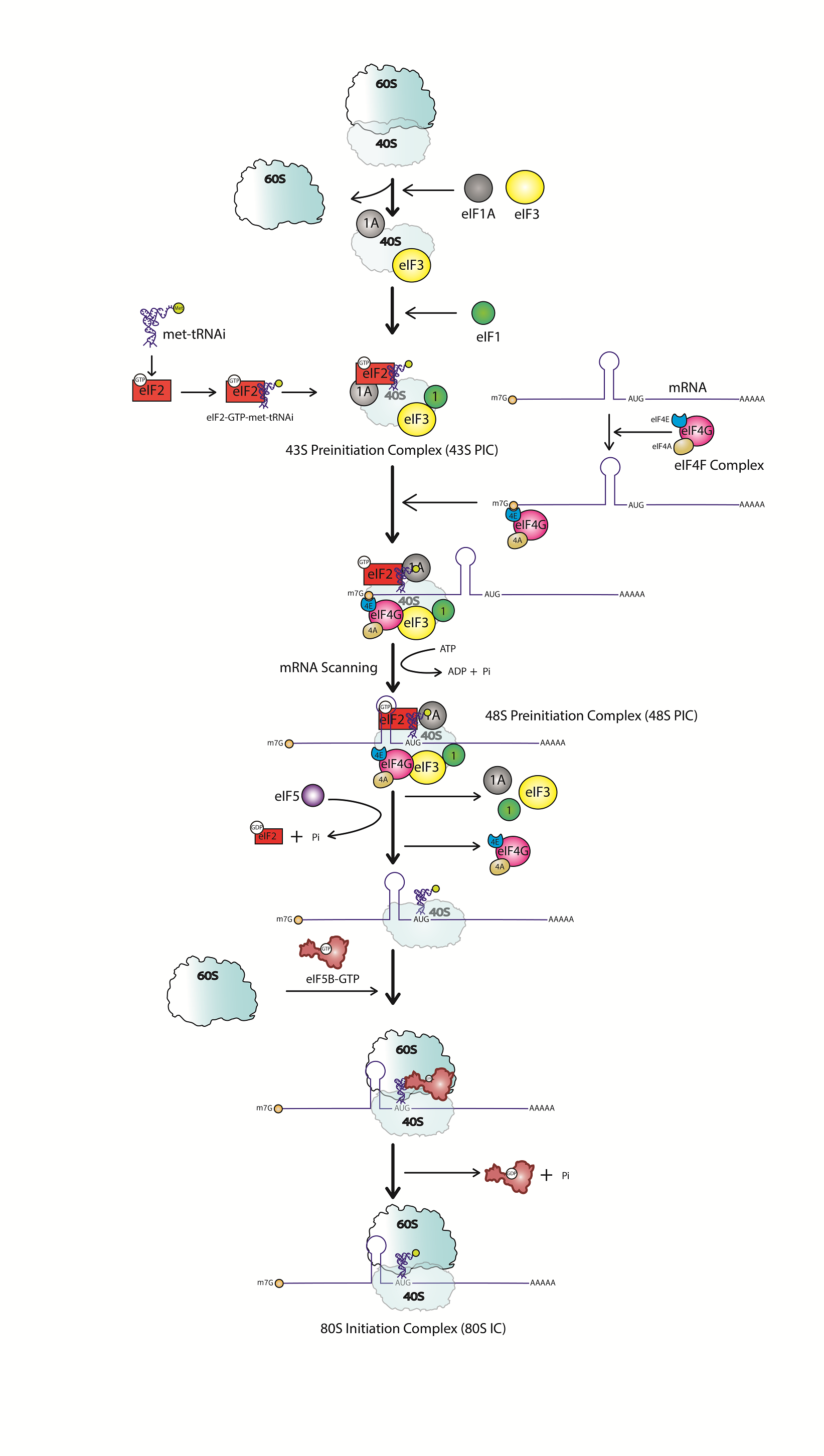

Supplement: Supplementary file 1 [file Image_1.TIF]
