# Supplementary material for: Established and Emerging Regulatory Roles of Eukaryotic Translation Initiation Factor 5B (eIF5B)
Source: Front Genet. 2021 Aug 27;12:737433. doi: 10.3389/fgene.2021.737433 (PMC8430213; doi:10.3389/fgene.2021.737433)
Supplement: Supplementary file 2 [file Data_Sheet_1.PDF]

Supplementary Figure 1

Overview of cap-dependent translation initiation:

During cap-dependent translation initiation, the interaction between eIF4E of eIF4F complex and 5′ 7-methyl guanosine (m7G) cap of mRNA helps in recruiting eIF4F complex onto the mRNA. Simultaneously, ternary complex (eIF2-GTP-met-tRNAi), eIF1 and eIF3 are recruited to the 40S subunit giving rise to 43S preinitiation complex (43S PIC). Ribosome is recruited onto the mRNA due to specific interaction between eIF4G (scaffold protein) and eIF3. Once recruited, mRNA is scanned by eIF4A (RNA helicase and ATP-dependent protein) until AUG start codon is encountered. After Start codon selection, eIF2 delivers Met-$tRNA_i^{Met}$ to the P-site of 40S subunit and a GTPase accelerating protein (GAP) eIF5 induces hydrolysis of GTP on eIF2. This leads to structural changes in the ribosome releasing most initiation factors. In the absence of eIF2, eIF5B stabilizes the Met-$tRNA_i^{Met}$ and simultaneously mediates 60S subunit association with 48S PIC leading to the formation of 80S initiation complex (80S IC). In the final step, GTP of eIF5B is hydrolyzed releasing remaining factors and 80S IC transitions into elongation phase.
